# Synergistic effect between the *KCNQ1* haplotype and alcohol consumption on the development of type 2 diabetes mellitus in Korean cohorts

**DOI:** 10.1038/s41598-021-01399-9

**Published:** 2021-11-08

**Authors:** Ji Young Park, Min-Gyu Yoo, Ji Ho Yun, Hye-Ja Lee, Sang Ick Park

**Affiliations:** Division of Endocrine and Kidney Disease Research, Department of Chronic Disease Convergence Research, Korea National Institute of Health, Korea Disease Control and Prevention Agency, 187 Osongsaengmyeong 2-ro, Osong-eup, Cheongju-si, Chungcheongbuk-do 28159 Republic of Korea

**Keywords:** Genetics, Biomarkers, Diseases

## Abstract

Potassium voltage-gated channel subfamily Q member 1 (*KCNQ1*) is one of the strongest susceptibility genes for type 2 diabetes mellitus (T2DM). Association studies between *KCNQ1* genetic variants and T2DM have been reported. The multifactorial disease T2DM is caused by interactions between genetic susceptibility and environmental factors. In this study, we examined the associations between the *KCNQ1* haplotype, which consists of the major alleles rs3852528, rs11024175, and rs2237892 (*ht*: ACC), and environmental factors such as alcohol consumption, which are related to the risk of T2DM, in two independent Korean populations. Data from health examination studies, i.e., HEXA (*n* = 50,357 subjects) and the Ansung–Ansan community-based Korean cohort study (*n* = 7603), were analyzed. In both cohorts, fasting blood glucose levels were significantly increased in moderate-to-heavy drinkers and carriers of the homozygous ACC haplotype. A significant association between the *KCNQ1* haplotype and alcohol consumption in the risk of diabetes was observed in the HEXA (OR 1.587; 95% CI 1.128–2.234) and Ansung–Ansan (OR 2.165; 95% CI 1.175–3.989) cohorts compared with abstainers not carrying the *KCNQ1* haplotype. Associations of the *KCNQ1* haplotype with alcohol consumption and β-cell function were observed in the Ansung–Ansan cohort. Moderate-to-heavy drinkers with the ACC haplotype had lower fasting insulin levels and mean 60 min insulinogenic index (IGI_60_) compared with light drinkers and abstainers not carrying the ACC haplotype. These findings indicate that *KCNQ1* variants play a synergistic role with alcohol consumption in the development of T2DM and impaired β-cell function.

## Introduction

Type 2 diabetes mellitus (T2DM) is a worldwide chronic metabolic disease that occurs when pancreatic β-cell function is impaired and insulin cannot be produced, leading to hyperglycemia^1,2^. T2DM is a multifactorial disease influenced by a combination of genetic and environmental factors.

One such environmental factor is alcohol consumption, which induces many problems in practical every aspect of human life. In particular, chronic alcohol consumption increases the risk of T2DM, leading to disruptions in glucose homeostasis and pancreatic β-cell function, including impaired insulin secretion and β-cell apoptosis^3,4^. Although the effects of alcohol consumption on the risk of T2DM have been reported by many studies, additional studies investigating the interplay between genetic variants and environmental factors leading to the onset of diabetes are needed.

Various susceptibility genes for T2DM have been identified by meta-analyses of genome-wide association studies (GWAS). Among these genes, the variants of voltage-gated potassium channel subfamily Q member 1 (*KCNQ1*), a novel diabetes susceptibility gene, have been implicated in diabetes in many studies in various ethnic groups^5–7^. *KCNQ1* encodes the pore-forming alpha subunit composed of the KvLQT1 (or Kv7.1) voltage-gated potassium channel and potassium voltage-gated channel subfamily E members (KCNEs)^8,9^. The KvLQT1 channel is essential for controlling the cellular repolarization process, including regulation of insulin secretion after electrical depolarization of pancreatic β-cells. Numerous *KCNQ1* variants are correlated with the prevalence of T2DM and the insulin response, along with increased fasting glucose levels resulting from impaired insulin secretion^10^. Despite that various *KCNQ1* single nucleotide polymorphisms (SNPs) are associated with susceptibility to T2DM, there are limited data on the association between the *KCNQ1* haplotype and T2DM. Furthermore, few studies have evaluated the interactions between genetic susceptibility and environmental risk factors in terms of T2DM risk. Here, we evaluated the multifactorial interplay between alcohol consumption and *KCNQ1* variants on the risk of developing T2DM in two independent Korean cohort studies: the HEXA (Health Examinees study) and Ansung–Ansan community-based Korean cohort study.

## Results

### General characteristics of the study population

The number of subjects with *KCNQ1* variants was similarly distributed between the two cohorts (Supplementary Table S1). The baseline characteristics of the subjects according to the *KCNQ1* haplotype, comprising the major alleles of three SNPs (*ht*: ACC/ACC), are shown in Table 1. Blood pressure and the levels of triglycerides, cholesterol (HDL and total), and the liver enzymes alanine aminotransferase and aspartate aminotransferase did not differ according to the *KCNQ1* haplotype in either cohort. The mean 60 min insulinogenic index (IGI_60_) values were 13.7 ± 29.6 in non-carriers, 12.4 ± 26.7 in ACC/– carriers, and 11.5 ± 19.3 in ACC/ACC carriers, indicating that subjects carrying the homozygous ACC haplotype (ACC/ACC carriers) tended to have decreased β-cell function in the Ansung–Ansan cohort (*p* < 0.0651). The fasting blood glucose level and prevalence rate of T2DM were significantly higher in subjects with the *KCNQ1* homozygous ACC haplotype than in non-carriers in both cohorts.

**Table 1:** General characteristics of the subjects (*KCNQ1* haplotype) in the HEXA and Ansung–Ansan cohorts.

	Non-carrier	ACC/–	ACC/ACC	*p*-value
**HEXA**				
Number of subjects (%)	11,669 (23.2)	25,782 (51.2)	12,906 (25.6)	
Age	53.5 ± 7.8	53.5 ± 7.8	53.4 ± 7.8	0.3992
Body mass index (kg/m^2^)	23.7 ± 2.6	23.6 ± 2.6	23.6 ± 2.6	0.7792
Systolic blood pressure (mmHg)	122.4 ± 14.8	122.2 ± 14.7	122.5 ± 14.9	0.1137
Diastolic blood pressure (mmHg)	75.8 ± 9.7	75.6 ± 9.7	75.8 ± 9.8	0.0516
Fasting glucose (mg/dL)	94.1 ± 19.5	94.8 ± 19.2	95.6 ± 19.5	< 0.0001
HDL-cholesterol (mg/dL)	54.1 ± 13.2	54.0 ± 13.2	54.0 ± 13.1	0.8930
Triglycerides (mg/dL)	125.2 ± 86.9	124.4 ± 85.5	125.9 ± 87.0	0.2623
Total cholesterol (mg/dL)	197.8 ± 35.2	197.8 ± 35.6	197.9 ± 35.9	0.9445
AST (IU/L)	24.0 ± 43.1	23.6 ± 12.8	23.4 ± 13.5	0.1583
ALT (IU/L)	22.0 ± 14.7	22.0 ± 14.7	21.9 ± 14.3	0.8818
Type 2 diabetes	1079 (9.3)	2585 (10.0)	1371 (10.6)	0.0015
Drinker (%)	5346 (45.8)	11,983 (46.5)	6004 (46.5)	0.4344
**Ansung–Ansan cohort**				
Number of subjects (%)	1747 (23.0)	3.842 (50.5)	2.014 (26.5)	
Age	51.6 ± 8.8	51.8 ± 8.8	51.8 ± 8.9	0.7610
Body mass index (kg/m^2^)	24.7 ± 3.0	24.5 ± 2.9	24.4 ± 3.0	0.0584
Systolic blood pressure (mmHg)	121.4 ± 18.7	121.5 ± 18.3	121.0 ± 18.7	0.6042
Diastolic blood pressure (mmHg)	80.5 ± 11.5	80.2 ± 11.4	79.9 ± 11.5	0.2159
Fasting glucose (mg/dL)	85.8 ± 15.4	88.0 ± 21.0	89.1 ± 23.5	< 0.0001
HDL-cholesterol (mg/dL)	44.5 ± 9.9	45.1 ± 10.1	45.0 ± 10.2	0.1367
Triglycerides (mg/dL)	162.0 ± 101.5	160.3 ± 105.2	163.2 ± 107.3	0.5731
Total cholesterol (mg/dL)	191.7 ± 35.4	192.1 ± 35.5	193.3 ± 36.4	0.3502
Fasting insulin (mg/dL)	7.7 ± 5.0	7.6 ± 4.5	7.5 ± 4.9	0.3361
IGI_60_	13.7 ± 29.6	12.4 ± 26.7	11.5 ± 19.3	0.0651
AST(IU/L)	29.9 ± 17.1	29.8 ± 20.8	29.5 ± 15.6	0.8588
ALT(IU/L)	28.0 ± 23.2	28.3 ± 32.8	27.8 ± 22.7	0.7382
Type 2 diabetes (%)	192 (11.0)	426 (11.1)	306 (15.2)	< 0.0001
Drinker (%)	895 (51.2)	1895 (49.3)	987 (49.0)	0.3261

All data except type 2 diabetes and alcohol consumption are presented as the mean ± standard deviation. One-way ANOVA was used for comparisons of continuous variables and the chi-square test for comparisons of categorical variables according to *KCNQ1* haplotype.

The baseline characteristics of the subjects according to their alcohol consumption status (27,024 abstainers and 23,333 drinkers in the HEXA cohort and 3826 abstainers and 3777 drinkers in the Ansung–Ansan cohort) are described in Supplementary Table S2. All drinkers had higher levels of alcoholic markers, including diastolic blood pressure, fasting glucose, HDL-cholesterol, triglycerides, aspartate aminotransferase, and alanine aminotransferase, compared with abstainers. Systolic blood pressure and the total cholesterol level differed significantly according to alcohol consumption only in the HEXA cohort (*p* < 0.001). The IGI_60_ value and fasting insulin level were lower in drinkers in the Ansung–Ansan cohort. The T2DM prevalence was not affected by alcohol consumption in either cohort; the prevalence rates of T2DM were 9.9% for abstainers and 10.1% for drinkers in the HEXA cohort and 11.8% for abstainers and 12.5% for drinkers in the Ansung–Ansan cohort.

### Effect of the association between the KCNQ1 haplotype and alcohol consumption on fasting blood glucose

The fasting glucose levels were increased in subjects carrying the *KCNQ1* haplotype compared with non-carriers, regardless of alcohol consumption, in the two cohort populations (Table 1). In addition, the fasting glucose levels were significantly higher in alcohol drinkers (Supplementary Table S2). We assessed the synergistic effect between alcohol consumption and the *KCNQ1* haplotype on the fasting glucose level (Table 2). In both cohorts, moderate-to-heavy drinkers and subjects carrying the ACC haplotype had higher fasting glucose levels than abstainers not carrying the ACC haplotype, and light alcohol consumption has shown increased fasting glucose levels regardless of ACC haplotype. Moderate-to-heavy drinkers with the carrying ACC haplotype had significantly high fasting glucose levels compared with abstainers carrying ACC haplotype.

**Table 2:** Effect of the *KCNQ1* haplotype on the fasting glucose level according to alcohol consumption in the HEXA and Ansung–Ansan cohorts.

	*KCNQ1*			*p-*value^1^
	Non-carrier	ACC/–	ACC/ACC	
**HEXA**				
Abstainers	93.0 ± 19.2	93.6 ± 18.3	94.4 ± 19.1	0.0002
Light	92.5 ± 16.1	93.3 ± 18.1	93.8 ± 16.9	0.0342
Moderate-to-heavy	94.4 ± 19.7	93.8 ± 20.2	99.5 ± 19.7	0.0078
*p-*value^2^	< 0.0001	< 0.0001	< 0.0001	
**Ansung–Ansan**				
Abstainers	83.7 ± 14.1	86.5 ± 21.0	87.4 ± 20.5	0.0001
Light	85.0 ± 15.3	86.2 ± 18.3	85.9 ± 15.7	0.5603
Moderate-to-heavy	87.4 ± 20.5	85.9 ± 15.7	93.7 ± 30.2	0.0031
*p-*value^2^	< 0.0001	< 0.0001	< 0.0001	

Data are expressed as the mean ± standard deviation fasting glucose level.

^1^One-way ANOVA was used to assess the relationships with the *KCNQ1* haplotype.

^2^Between alcohol consumption.

### The effects of the KCNQ1 haplotype and alcohol consumption on T2DM risk

Multivariate logistic regression analysis was performed to assess the association between the *KCNQ1* haplotype and alcohol consumption in the risk of T2DM (Table 3). In the HEXA cohort, light drinkers carrying the ACC haplotype exhibited a higher risk of developing T2DM (odds ratio [OR] 1.530; 95% confidence interval [CI] 1.008–2.323) and the T2DM risk was significantly increased in moderate-to-heavy drinkers carrying ACC haplotype (OR 1.587; 95% CI 1.128–2.234) compared with abstainers not carrying the *KCNQ1* ACC haplotype, whereas the T2DM risk was not higher in abstainers carrying the ACC haplotype (OR 1.334; 95% CI 0.888–2.003) compared with non-carriers. Also, ACC haplotype heterozygotes and light drinking had risky effects on T2DM in HEXA population. In Ansung–Ansan, subjects carrying the homozygous ACC haplotype showed an increased risk of T2DM (OR 2.588 in abstainers; 95% CI 1.498–4.469; OR 2.165; 95% CI 1.175–3.989 in moderate-to-heavy drinkers). In both cohorts, the risk of T2DM was increased in moderate-to-heavy drinkers who were homozygous for the ACC haplotype compared with abstainers not carrying the ACC haplotype. As shown in Supplementary Table S3, the risk of T2DM showed a similar pattern between subjects carrying the major *KCNQ1* SNP alleles and carrying the ACC haplotype. Based on the *KCNQ1* genetic factor, which had a more significant effect on the risk of T2DM than environmental factors, subjects with the major alleles of each 3 SNPs and the ACC haplotype had a higher T2DM risk compared with those who did not have the major alleles or the haplotype. These results indicate a significant increase in T2DM risk by the *KCNQ1* haplotype and alcohol consumption in HEXA, but the ACC haplotype was a little more influential factor in the Ansung–Ansan cohort.

**Table 3:** Association between type 2 diabetes and the *KCNQ1* haplotype according to alcohol consumption in the HEXA and Ansung–Ansan cohorts.

	*KCNQ1* (OR, 95% CI)		
	Non-carrier	ACC/–	ACC/ACC
**HEXA**			
Abstainers	Ref	1.232 (0.858–1.769)	1.334 (0.888–2.003)
Light	1.265 (0.816–1.961)	1.511 (1.049–2.177)	1.530 (1.008–2.323)
Moderate-to-heavy	1.247 (0.879–1.768)	1.452 (1.047–2.012)	1.587 (1.128–2.234)
**Ansung–Ansan cohort**			
Abstainers	Ref	1.659 (0.982–2.800)	2.588 (1.498–4.469)
Light	0.514 (0.170–1.551)	1.198 (0.617–2.327)	1.712 (0.829–3.535)
Moderate-to-heavy	1.650 (0.870–3.129)	1.587 (0.892–2.826)	2.165 (1.175–3.989)

The *p*-values were calculated by multivariate logistic regression models adjusted for age, sex, smoking, body mass index, family history of diabetes, physical activity, and income level.

### Effect of the association between the KCNQ1 haplotype and alcohol consumption on β-cell function

As shown in Table 4, the fasting insulin levels decreased consistently with the result of fasting glucose levels by carrying the ACC haplotype and alcohol consumption in Ansung–Ansan cohort. We determined the insulin index on the subjects, excepted patients with T2DM, related to the fasting glucose level, fasting insulin level, IGI_60_, homeostatic model assessment for insulin resistance, and the insulin sensitivity according to the *KCNQ1* haplotype and alcohol consumption to understand the effect on increased fasting glucose levels. Alcohol drinking showed significantly lower fasting insulin levels according to alcohol exposure amount than abstainers. Also, subjects carrying ACC haplotype showed decreased tendency of fasting insulin levels. We detected the association effects between alcohol consumption and carrying *KCNQ1* haplotype on the reduction of fasting insulin levels. The IGI_60_ value tended to be lower in those carrying the ACC haplotype than in non-carriers. Also, drinkers showed a lower the IGI_60_ value than abstainers regardless of alcohol consumption amount. The IGI_60_ value was increased in light drinkers compared to moderate-to-heavy drinkers and abstainer carrying ACC haplotype, but not significant. Additionally, many studies have reported an importance of insulin sensitivity than insulin secretion, but there were no significant differences in the homeostatic model assessment for insulin resistance or insulin sensitivity index in the present study (Supplementary Table S4). Previous studies have consistently reported that alcohol consumption contributes to impaired insulin secretion, as found in the present study (Supplementary Table S2). Our results suggest that the interplay between the *KCNQ1* ACC haplotype and alcohol consumption increases the risk of T2DM resulting from pancreatic β-cell dysfunction, rather than a change in insulin resistance and/or insulin sensitivity.

**Table 4:** Changes in the fasting insulin level and β-cell function according to the *KCNQ1* haplotype and alcohol consumption in the Ansung–Ansan cohort.

	Non-carrier	ACC/–	ACC/ACC		*p* for trend
**Fasting insulin**					
Abstainers	8.1 ± 5.6	7.8 ± 4.4	7.7 ± 4.6	0.2239	< 0.0001
Low	7.6 ± 4.8	7.5 ± 4.8	7.4 ± 5.1	0.8429	
Moderate-to-heavy	7.1 ± 4.0	7.2 ± 3.9	7.0 ± 5.1	0.7472	
*p-*value	0.0037	0.0015	0.0218		
**IGI**_**60**_					
Abstainers	15.0 ± 36.5	12.8 ± 27.4	12.2 ± 19.9	0.1325	0.0066
Low	14.2 ± 22.9	13.8 ± 29.0	12.7 ± 19.1	0.7819	
Moderate-to-heavy	12.3 ± 21.5	11.3 ± 17.4	10.8 ± 19.6	0.4653	
*p-*value	0.3513	0.1459	0.3671		

Data are expressed as the mean ± standard deviation. *IGI*_*60*_ insulinogenic index at 1 h after the oral glucose tolerance test. The participants with T2DM were excluded in cohort panel. Differences in the association between the *KCNQ1* haplotype and alcohol consumption were assessed by one-way ANOVA.

## Discussion

In the present study, we found an effect of the association between alcohol consumption and *KCNQ1* variants on the development of T2DM in two independent cohorts. Drinkers carrying the ACC haplotype showed an increased risk of T2DM and higher fasting glucose levels due to impaired insulin secretion compared with abstainers not carrying the ACC haplotype.

A GWAS conducted by an independent study group identified several SNPs associated with susceptibility to T2DM^5,7,11^. Notably, *KCNQ1* SNPs were correlated with the incidence of T2DM in two independent GWAS of Japanese populations. Furthermore, various SNPs in *KCNQ1* have been associated with T2DM susceptibility regardless of ethnicity. *KCNQ1* rs2237892, which alters pancreatic β-cell function, is the most well-known susceptibility SNP for T2DM and gestational diabetes mellitus in the Asian population^12^. The high-risk SNPs rs151290, rs2237892, and rs2237895 have been associated with T2DM due to impaired glucose-stimulated insulin secretion and higher LDL and total cholesterol levels^10^. The major *KCNQ1* SNPs (rs2237897, rs2237892, and rs2283228) showed significant associations with a higher fasting glucose level and reduced insulin response^7^. Homozygosity for the CC risk allele of rs2237892 was associated with a significantly lower homeostatic model assessment of β-cell function (HOMA-β) and corrected insulin response compared with the other two genotypes. Similar to our findings, individuals with the *KCNQ1* variant were susceptible to diabetes in two independent cohorts.

We found that subjects carrying the ACC haplotype had the major alleles rs3852528, rs2237892, and rs1102417, which are independently associated with the risk of developing T2DM. Riyadh et al*.* reported that the *KCNQ1* haplotype consists of the minor alleles rs2237892, rs2283228, and rs2237895, which have a protective effect on T2DM via a homozygous TCA haplotype (TCA-TCA diplotype). *KCNQ1* exhibits improved β-cell function, as reflected by a higher HOMA-β value, compared with the other diplotypes^6^. Consistent with our genetic analysis, carrying the homozygous ACC haplotype results in a higher T2DM prevalence and lower IGI_60_ value.

Carrying the *KCNQ1* haplotype and alcohol consumption increase the effect of the fasting glucose level on β -cell dysfunction, leading to an increased risk of T2DM. Several studies have reported that chronic heavy alcohol consumption impairs β-cell function, leading to a higher risk of T2DM^13,14^. Although the effect of alcohol intake according to exposure amount and period on the development of T2DM is still controversial, such as J- and linear-curve, alcohol consumption is thought to be a major determinant of T2DM development^15,16^. In this study, we detected the effects of alcohol consumption on the regulation of the fasting glucose level and insulin secretion. Alcohol intake tended to have linear relation to the increase of fasting glucose by *KCNQ1* haplotype carrying in HEXA study, but not in Ansung–Ansan study (Table 2). Also, Moderate-to-heavy drinkers having *KCNQ1* haplotype homozygotes showed higher association with T2DM risk in both HEXA and Ansung–Ansan cohorts (Table 3). Moreover, *KCNQ1* haplotype carriers had a tendency to lower fasting insulin and IGI_60_ level according to the higher intake of alcohol in Ansung–Ansan study (Table 4). Therefore, the combination of alcohol consumption and carrying *KCNQ1* variants increased the risk of developing T2DM and gradually increased fasting glucose levels with β-cell dysfunction.

KCNQ1 combined with KCNE1 is responsible for positioning the KvLQT1 channel in pancreatic β-cells. The KvLQT1 channel is activated by electrical depolarization resulting from closure of the K_ATP_ channel, and it induces subsequent repolarization, leading to termination of insulin secretion via an outwardly opening K^+^ channel, to regulate ion homeostasis^8,17,18^. A specific deficiency of *KCNQ1* in pancreatic β-cells and loss of function of the KvLQT1 channel are associated with prolonged insulin secretion via inhibition of action potential repolarization^19^. Rapid repolarization, including KvLQT1 gain of function, interrupts insulin secretion, and blocking the KCNQ1 subunit promotes insulin secretion by inhibiting after-repolarization of action potentials in pancreatic β-cells. Although these variants have not been reported to induce a functional effect on the KvLQT1 channel^20^, the *KCNQ1* polymorphisms rs3852528, rs11024175, and rs2237892, which are located in intron 15 of the *KCNQ1* gene on chromosome 11p15, have been reported as susceptible variants to T2DM. The haplotype analyses including these each single variants may reflect more effectively genetic heritability of *KCNQ1* on T2DM than those of individual risky variants. Furthermore, many studies have reported correlations of T2DM with impaired β-cell function and the *KCNQ1* variant located in the intron. Even if a mutation in that intron did not affect the KvLQT1 channel, several cases affect the functional consequences that the novel mutation in the third base of intron 7 (c.1003G > A) is a splicing-related mutation within the pore domain S6 region of the KvLQT1 channel^21^. In addition, *KCNQ1* variants located in intron 15, such as rs2274196 and rs163184, may induce subsequent changes in gene expression and binding activity. The SNP rs163184 locus is contiguous to rs2237892 and rs2237895, which moderates linkage disequilibrium. The SNP rs163184 locus is contiguous to rs2237892 and rs2237895, which are in moderate linkage disequilibrium with each other^22^. Overall, these findings suggest that the *KCNQ1* variants associated with the ACC haplotype affect the expression of other target genes and/or the transcription of the nearby imprinting region, encompassing *CDKN1C*, *PHLDA2*, and *Slc22a18,* which are related to β-cell proliferation^23–26^. Based on our results, further studies are required to validate the correlation and to investigate the effects of *KCNQ1* variants within introns that potentially regulate β-cell proliferation or a functional modification of the KvLQT1 channel.

## Materials and methods

### Study population

Data were obtained from two cohort studies conducted by the Korea National Institutes of Health as part of the Korean Genome and Epidemiology study^27^. Written informed consent was obtained from all subjects, and this research project was approved by the National Biobank of Korea and the Centers for Disease Control and Prevention, Republic of Korea. This study protocol was approved by the Korean National Institutes of Health Institutional Review Board (2019-03-01-PE-A). All study protocols were carried out in accordance with approved guidelines.

Participants of the HEXA were enrolled from 38 health examination centers and hospitals located in eight regions (metropolitan areas or major cities) of Korea between 2004 and 2013. We used baseline data from HEXA (n = 53,754). Subjects with missing data on alcohol consumption (n = 2924) or the *KCNQ1* haplotype (n = 473) were excluded. A total of 50,357 subjects were included in this study. The community-based Ansung–Ansan cohort study has been described in detail previously^28^. We used data from 8840 subjects living in rural Ansung or urban Ansan. Individuals with missing data on alcohol consumption (n = 828) or type 2 diabetes (n = 409) were excluded, leaving 7603 subjects who were included in this study.

### Anthropometric and biochemical analyses

Information on age, family history of diabetes, physical activity, and smoking (pack-years) was obtained using interview-based questionnaires. Body mass index (BMI) was calculated as weight in kilograms divided by height in meters squared. Fasting glucose levels and the lipid profile (total cholesterol, triglycerides, and HDL-cholesterol) were measured using the Hitachi 747 chemistry analyzer (Hitachi Ltd., Tokyo, Japan) following the manufacturer’s recommendations. Insulin levels were measured using the INS-IRMA kit (Biosource, Nivelles, Belgium) and a gamma counter (Packard Instrument Co., Meriden, CT, USA) in the Ansan–Ansung cohort, whereas insulin levels were not measured in the HEXA cohort.

### Genotyping

All genotype data were approved and provided by the National Biobank of Korea and the Centers for Disease Control and Prevention, the Republic of Korea (no. 2019-022). Genotyping in the Ansung–Ansan cohort study was performed using the Affymetrix genome-wide human SNP array 5.0, and genotype imputation was performed using IMPUTE2; the 1000 genome projects of the East Asian ancestry sample was used as a reference panel. Genotyping in the HEXA study was performed using the Korean Chip designed by the Center for Genome Science at the Korean National Institutes of Health. The expected genotyping and quality-control information has been described previously in detail^29,30^.

### T2DM and pancreatic β-cell function

T2DM was defined as a fasting glucose level > 126 mg/dL. In addition, subjects who reported currently taking antidiabetic medication and insulin were considered to have T2DM. The subjects were followed until they developed T2DM or the last examination. In the Ansung–Ansan cohort, pancreatic β-cell function was estimated using the oral glucose tolerance test (75 g). The mean 60 min insulinogenic index (IGI_60_) was calculated as follows: (insulin level [µU/mL]) at 60 min–insulin level at 0 min) ÷ (glucose level [mmol/L] at 60 min–glucose level at 0 min)^13^.

### Measurement of alcohol consumption

Information on alcohol consumption was collected from the two cohorts using an interview-based questionnaire. Subjects were asked whether they had ever consumed at least one alcoholic drink per month; if yes, they were then asked whether they were former or current drinkers. We excluded former drinkers because the drinking period was unclear. Because females were not in the heavy drinking group, we classified the subjects into three groups (abstainers, light, and moderate-to-heavy drinkers) using the baseline alcohol consumption information.

### Statistical analysis

Statistical analyses were performed using the SAS software package (ver. 9.4; SAS Institute, Cary, NC, USA). Data are presented as means ± standard deviation, numbers (%), or odds ratios (ORs) with 95% confidence intervals (CIs). Logarithmic transformation was applied to variables with a non-Gaussian distribution. Student’s *t*-test and One-way ANOVA analysis was conducted to compare the clinical characteristics according to drinking status. The chi-square test was used to compare categorical variables (drinking status and the *KCNQ1* haplotype). Multivariate logistic regression analyses were performed to detect the association between T2DM and the *KCNQ1* haplotype according to alcohol consumption in the HEXA and Ansan–Ansung cohorts after adjusting for age, sex, smoking, BMI, family history of diabetes, physical activity, and income. All reported *p*-values are two-tailed, and a *p*-value < 0.05 was considered significant.

## Conclusion

In summary, we determined the characteristics of the *KCNQ1* ACC haplotype and the synergistic effect between the *KCNQ1* haplotype and alcohol consumption on the increased fasting glucose level and the risk of T2DM along with β-cell dysfunction. This is the first study to examine the effect of the association between *KCNQ1* and alcohol consumption on the risk of developing T2DM. However, our study also had some limitations. First, the mechanism of how the *KCNQ1* intronic variants interact with alcohol consumption and T2DM remains unclear. Second, we used IGI_60_ as the index of insulin secretion because 30 min glucose and insulin values were not available. But, the insulinogenic index at 60 min correlates well with IGI_30_ and can be used as a surrogate of early insulin secretion^31^. Furthermore, the present study has several strengths, such as the use of a large-scale sample size and replicated results using clinical indicators (glucose and insulin) to identify T2DM in two independent cohorts. These findings potentially suggest that the interaction between the *KCNQ1* variants and alcohol consumption contributes to the pathogenesis of T2DM via pancreatic β-cell dysfunction.

## Supplementary Information

Supplementary Information.
