# Supplementary material for: Synergistic effect between the KCNQ1 haplotype and alcohol consumption on the development of type 2 diabetes mellitus in Korean cohorts
Source: Sci Rep. 2021 Nov 8;11:21796. doi: 10.1038/s41598-021-01399-9 (PMC8575903; doi:10.1038/s41598-021-01399-9)
Supplement: Supplementary file 1 — Supplementary Information. [file 41598_2021_1399_MOESM1_ESM.docx]

**Synergistic effect between the *KCNQ1* haplotype and alcohol consumption on the development of type 2 diabetes mellitus in Korean cohorts**

Ji Young Park ^1, †^, Min-Gyu Yoo ^1, †^, Ji Ho Yun ^1^, Hye-Ja Lee^1, *^ and Sang Ick Park^1, *^

Division of Endocrine and Kidney Disease Research, Department of Chronic Disease Convergence Research, Korea National Institute of Health, Korea Disease Control and Prevention Agency, 187 Osongsaengmyeong 2-ro, Osong-eup, Cheongju-si, Chungcheongbuk-do, 28159, Republic of Korea

Ji Young Park and Min-Gyu Yoo contributed equally.

^†^**These authors contributed equally as co-first authors**

^*^**Correspondence to:**

Hye-Ja Lee, PhD

Phone: +82-43-719-8692, Fax: +82-43-719-8602, Email: [hyejalee@yahoo.co.kr](mailto:hyejalee@yahoo.co.kr)

Sang Ick Park, PhD

Phone: +82-43-719-8690, Fax: +82-43-719-8602, Email: [parksi61@hotmail.com](mailto:parksi61@hotmail.com)

|  | Major | Hetero | Minor |
| --- | --- | --- | --- |
| **HEXA** |  |  |  |
| rs3852528 (A/G) | 15,698 (31.2) | 25,022 (49.7) | 9,637 (19.1) |
| rs2237892 (C/T) | 19,550 (38.8) | 23,782 (47.2) | 7,025 (14.0) |
| rs11024175 (C/T) | 16,222 (32.2) | 24,916 (49.5) | 9,219 (18.3) |
| **Ansung-Ansan** |  |  |  |
| rs3852528 (A/G) | 2,417 (31.8) | 3,749 (49.3) | 1,437 (18.9) |
| rs2237892 (C/T) | 2,904 (38.2) | 3,572 (47.0) | 1,127 (14.8) |
| rs11024175 (C/T) | 2,438 (32.1) | 3,751 (49.3) | 1,414 (18.6) |

**Table S1. Frequencies of *KCNQ1* SNPs in the two study cohorts.**

|  | Abstainers | Drinkers | *p*-value |
| --- | --- | --- | --- |
| **HEXA** |  |  |  |
| Number of subjects (%) | 27,024 (53.7) | 23,333 (46.3) |  |
| Age | 54.3 ± 7.6 | 52.5 ± 7.9 | <0.0001 |
| Body mass index (kg/m^2^) | 23.7 ± 2.9 | 24.0 ± 2.8 | <0.0001 |
| Systolic blood pressure (mmHg) | 121.4 ± 14.9 | 123.3 ± 14.6 | <0.0001 |
| Diastolic blood pressure (mmHg) | 74.8 ± 9.6 | 76.8 ± 9.8 | <0.0001 |
| Fasting glucose (mg/dL) | 93.6 ± 18.7 | 96.2 ± 20.0 | <0.0001 |
| HDL-cholesterol (mg/dL) | 53.9 ± 12.8 | 54.2 ± 13.6 | 0.0026 |
| Triglycerides (mg/dL) | 118.3 ± 74.6 | 132.8 ± 97.6 | <0.0001 |
| Total cholesterol (mg/dL) | 198.8 ± 35.9 | 196.8 ± 35.2 | <0.0001 |
| AST (IU/L) | 23.3 ± 29.9 | 24.1 ± 13.1 | <0.0001 |
| ALT (IU/L) | 21.4 ± 25.8 | 23.2 ± 18.7 | <0.0001 |
| Type 2 diabetes | 2,699 (9.9) | 2,382 (10.1) | 0.4298 |
| KCNQ1 haplotype |  |  | 0.4344 |
| ACC carrier | 6,902 (25.5) | 6,004 (25.7) |  |
| ACC/- | 13,799 (51.1) | 11,983 (51.4) |  |
| Non-carrier | 6,323 (23.4) | 5,346 (22.9) |  |
| **Ansung-Ansan** |  |  |  |
| Number of subjects (%) | 3,826 (50.3) | 3,777 (49.7) |  |
| Age | 53.3 ± 9.0 | 50.2 ± 8.4 | <0.0001 |
| Body mass index (kg/m^2^) | 24.7 ± 3.2 | 24.5 ± 3.0 | 0.0008 |
| Systolic blood pressure (mmHg) | 121.6 ± 19.2 | 121.1 ± 17.7 | 0.3268 |
| Diastolic blood pressure (mmHg) | 79.3 ± 11.4 | 81.2 ± 11.4 | <0.0001 |
| Fasting glucose (mg/dL) | 86.1 ± 19.5 | 89.4±21.5 | <0.0001 |
| HDL-cholesterol (mg/dL) | 44.1 ± 9.8 | 45.8 ± 10.3 | <0.0001 |
| Triglycerides (mg/dL) | 152.0 ± 88.8 | 171.0 ± 118.3 | <0.0001 |
| Total cholesterol (mg/dL) | 191.8 ± 35.1 | 192.8 ± 36.4 | 0.2207 |
| Fasting insulin (mg/dL) | 7.9 ± 4.8 | 7.3 ± 4.6 | <0.0001 |
| IGI_60_ | 13.1 ± 29.5 | 11.7 ± 21.0 | 0.0377 |
| AST (IU/L) | 27.8 ± 18.2 | 31.7 ± 19.0 | <0.0001 |
| ALT (IU/L) | 25.6 ± 31.9 | 30.7 ± 23.9 | <0.0001 |
| Type 2 diabetes | 452 (11.8) | 472 (12.5) | 0.3623 |
| KCNQ1 haplotype |  |  | 0.3261 |
| ACC carrier | 1,027 (26.8) | 987 (26.1) |  |
| ACC/- | 1,947 (50.9) | 1.895 (50.2) |  |
| Non-carrier | 852 (22.3) | 895 (23.7) |  |

**Table S2. Baseline characteristics according to alcohol consumption in the HEXA and Ansung-Ansan cohorts.** All data except type 2 diabetes and alcohol consumption are presented as the mean ± standard deviation. Participants who did not consume alcohol were classified as abstainers and those who consumed alcohol as drinkers. Student’s *t*-test and the chi-square test were used to determine differences in the variables according to alcohol consumption.

|  | *KCNQ1* (OR, 95% CI) | | |
| --- | --- | --- | --- |
|  | **Major** | **Hetero** | **Minor** |
| **HEXA** |  |  |  |
| rs3852528 |  |  |  |
| Abstainers | 1.483(0.972-2.263) | 1.297(0.870-1.933) | Ref |
| Low | 1.675(1.089-2.576) | 1.569(1.049-2.348) | 1.403(0.863-2.282) |
| Moderate-to-heavy | 1.755(1.209-2.548) | 1.492(1.035-2.152) | 1.357(0.917-2.008) |
| rs2237892 |  |  |  |
| Abstainers | 1.466(0.919-2.338) | 1.314(0.829-2.081) | Ref |
| Low | 1.816(1.135-2.906) | 1.573(0.990-2.499) | 1.185(0.651-2.155) |
| Moderate-to-heavy | 1.864(1.211-2.869) | 1.510(0.982-2.322) | 1.114(0.694-1.790) |
| rs11024175 |  |  |  |
| Abstainers | 1.139(0.758-1.712) | 1.035(0.704-1.523) | Ref |
| Low | 1.425(0.942-2.154) | 1.220(0.826-1.800) | 1.247(0.775-2.007) |
| Moderate-to-heavy | 1.448(1.014-2.068) | 1.236(0.871-1.753) | 1.067(0.730-1.560) |
| **Ansung-Ansan** |  |  |  |
| rs3852528 |  |  |  |
| Abstainers | 2.985(1.572-5.668) | 2.272(1.213-4.257) | Ref |
| Low | 2.255(1.034-4.919) | 1.433(0.672-3.057) | 0.606(0.166-2.206) |
| Moderate-to-heavy | 2.736(1.375-5.447) | 1.998(1.017-3.927) | 2.163(1.028-4.553) |
| rs2237892 |  |  |  |
| Abstainers | 2.516(1.303-4.856) | 1.727(0.893-3.338) | Ref |
| Low | 1.620(0.736-3.567) | 1.310(0.602-2.851) | 0.413(0.089-1.912) |
| Moderate-to-heavy | 2.240(1.112-4.512) | 1.728(0.858-3.480) | 1.614(0.714-3.649) |
| rs11024175 |  |  |  |
| Abstainers | 2.566(1.403-4.692) | 1.852(1.024-3.348) | Ref |
| Low | 1.867(0.882-3.953) | 1.235(0.597-2.554) | 0.522(0.146-1.861) |
| Moderate-to-heavy | 2.750(1.422-5.247) | 1.452(0.760-2.774) | 1.872(0.914-3.837) |

**Table S3. The risk of developing T2DM according to *KCNQ1* SNPs and alcohol consumption in the HEXA and Ansung-Ansan cohorts.** The *p*-values were calculated by multivariate logistic regression models adjusted for age, sex, smoking, body mass index, family history of diabetes, physical activity, and income level.

|  | | Abstainers | | | Drinkers | | | *p*-value |
| --- | --- | --- | --- | --- | --- | --- | --- | --- |
|  | Non-carrier | | ACC/- | ACC/ACC | Non-carrier | ACC/- | ACC/ACC |  |
| HOMA-IR | | 1.7±1.2 | 1.7±1.1 | 1.7±1.1 | 1.6±1.0 | 1.7±1.2 | 1.6±1.2 | 0.2522 |
| ISI | | 10.2±9.2 | 10.2±8.6 | 10.9±20.8 | 11.0±8.9 | 10.8±9.0 | 10.9±8.8 | 0.2224 |

**Table S4. Changes in insulin resistance and insulin sensitivity according to genetic KCNQ1 haplotype and alcohol consumption in Ansung-Ansan cohort.**

Difference among the genotype-alcohol consumption were assessed by One-Way ANOVA analysis.
